# Metformin: A New Inhibitor of the Wnt Signaling Pathway in Cancer

**DOI:** 10.3390/cells12172182

**Published:** 2023-08-30

**Authors:** Domenico Conza, Paola Mirra, Francesca Fiory, Luigi Insabato, Antonella Nicolò, Francesco Beguinot, Luca Ulianich

**Affiliations:** 1 URT Genomics of Diabetes, Institute of Endocrinology and Experimental Oncology, National Research Council & Department of Translational Medicine, University of Naples “Federico II”, 80131 Naples, Italy; domenico.conza@libero.it (D.C.); paolamirra06@gmail.com (P.M.); francesca.fiory@unina.it (F.F.); antonellan43@gmail.com (A.N.); beguino@unina.it (F.B.); 2 Department of Advanced Biomedical Sciences, University of Naples “Federico II”, 80131 Naples, Italy; insabato@unina.it

**Keywords:** metformin, cancer, Wnt

## Abstract

The biguanide drug metformin is widely used in type 2 diabetes mellitus therapy, due to its ability to decrease serum glucose levels, mainly by reducing hepatic gluconeogenesis and glycogenolysis. A considerable number of studies have shown that metformin, besides its antidiabetic action, can improve other disease states, such as polycystic ovary disease, acute kidney injury, neurological disorders, cognitive impairment and renal damage. In addition, metformin is well known to suppress the growth and progression of different types of cancer cells both in vitro and in vivo. Accordingly, several epidemiological studies suggest that metformin is capable of lowering cancer risk and reducing the rate of cancer deaths among diabetic patients. The antitumoral effects of metformin have been proposed to be mainly mediated by the activation of the AMP-activated protein kinase (AMPK). However, a number of signaling pathways, both dependent and independent of AMPK activation, have been reported to be involved in metformin antitumoral action. Among these, the Wingless and Int signaling pathway have recently been included. Here, we will focus our attention on the main molecular mechanisms involved.

## 1. Introduction

Metformin, a biguanide drug, is the most prescribed oral antidiabetic agent worldwide, taken by over 150 million people annually [1]. It is able, indeed, to lower the plasma glucose level mainly by inhibiting hepatic gluconeogenesis (HGP) and improving insulin resistance with negligible hypoglycemia side effects [2,3]. Many of both the systemic indirect and direct effects exerted by metformin are thought to be mediated through the activation of the Adenosine monophosphate (AMP)-activated protein kinase (AMPK), a regulator of energy metabolism induced by cellular stresses that cause a depletion of cellular ATP (Adenosine triphosphate) content, thus increasing the AMP/ATP ratio [4,5,6]. It has been shown, indeed, that the inhibition of AMPK signaling significantly reduces the efficiency of metformin in the treatment of type 2 diabetes mellitus (T2DM) and atherosclerosis [7]. Once activated, AMPK can inhibit the mammalian target of rapamycin (mTOR), thereby regulating different pathways involved in glucose, lipid and energy metabolism. Besides T2DM, metformin has been reported to be effective in the treatment of other diseases such as nephropathy [8], polycystic ovary syndrome [9], neurological disorders [10] and cardiovascular diseases [11], which are often associated with insulin resistance or diabetes. Evans and coworkers were the first to recognize the antitumor properties of metformin in 2005 [12]. They found, indeed, an inverse correlation between cancer occurrence and metformin treatment in diabetic patients, launching the investigation on the usage of metformin and other biguanides in both cancer therapy and prevention. Since then, a plethora of studies have been performed that support the antitumoral properties of metformin in different cancer types where diabetes represents an important risk factor, such as kidney, pancreas, liver, lung, non-Hodgkin lymphomas, prostate, rectum, endometrial, breast, and colon cancers [13,14]. In these cases, metformin reduces both the risk of cancer and the rate of cancer deaths [15]. As for diabetes, the anticancer properties of metformin are thought to be due to the activation of AMPK and the consequent inhibition of mTOR, leading to protein synthesis inhibition and, thus, decreasing the proliferation of cancer cells. However, although a considerable number of effects have been described, the mechanisms of action underlying the antitumoral activity of metformin remain only partially elucidated and it is becoming increasingly clear that metformin can modulate different molecular pathways [16]. Several studies have recently reported that the Wing-less and Int (Wnt) signaling pathway can be affected by metformin. This pathway regulates embryonic development and different physiologic functions such as stem cell regulation but also cell migration, wound healing, and maintenance of tissue homeostasis [17]. However, it plays a key role also in cancer, favoring the initiation, progression, and invasion of cancer cells [17]. Thus, a better understanding of the molecular mechanism through which metformin can modulate the Wnt pathway might be extremely relevant in the perspective of a therapeutic use of metformin, alone or in association with other antineoplastic agents, especially in those tumors in which this pathway is deregulated. Here, we will provide an overview of the main molecular mechanisms implicated in the Wnt-related anticancer effects of metformin.

## 2. The Wnt Signaling Pathway

### 2.1. The Canonical Wnt Signaling Pathway

The Wnt/β-catenin signaling pathway is traditionally known as the “canonical” Wnt pathway. It plays a crucial role in development and in many physiological aspects, but it also drives pivotal processes in cancer such as progression, invasion, metastasis, and drug resistance in hematological malignancies and solid tumors [17,18]. Under normal physiological conditions, the transcription factor β-catenin is destroyed by the β-catenin destruction complex formed by adenomatous polyposis (APC), casein kinase I (CK I), glycogen synthase kinase 3β (GSK3β) and Axin [19,20,21]. Axin works as a scaffolding protein whether β-catenin is phosphorylated by CK I and GSK3β in different residues [19,20,21]. At this point, the protein is degraded by the E3 ubiquitin ligase (β-TrCP) [22]. However, the binding of the Wnt protein ligands Wnt1, Wnt2, Wnt3, Wnt3a, Wnt8a, Wnt8b, Wnt10a, and Wnt10b to the complex formed by the cell membrane Frizzled protein (Fz) and the low-density lipoprotein receptor-associated protein (LRP5/6), changes the conformation of the complex itself, leading to LRP phosphorylation and to the association of the cytoplasmic disheveled proteins (DVL) to Fz, increasing the binding of Axin to heterodimers [23]. In turn, the stability of the degradation complex is decreased and the phosphorylation of β-catenin by CK1 and by unphosphorylated GSK-3β is blocked, as well as the targeting of the protein for ubiquitination and proteasome degradation. The result is the accumulation of unphosphorylated β-catenin in the cytoplasm and its translocation to the nucleus [24], where binding to the T-cell transcription factor/lymphoid enhancer-binding factor (TCF/LEF) occurs. This interaction, finally, determines the transcriptional upregulation of downstream target genes Axin2, c-Myc, Cyclin D1, Survivin, Matrix Metalloproteinases (MMPs) and others [25,26,27]. Wnt antagonists have been divided into two classes, characterized by different molecular mechanisms: the first class includes proteins able to bind Wnt ligands, such as Cerberus, Wnt inhibitory factor-1 (WIF-1), and secreted Frizzled-related proteins (SFRPs); the second class includes proteins able to bind Lrp5/6 and that are constituents of the Wnt receptor complex Dickkopf (Dkk) [28]. The canonical Wnt pathway has the capability to regulate the epithelial–mesenchymal transition (EMT), a process that mediates the invasion and progression of tumors [29]. The connections between E-cadherin and β-catenin play a key role in this process: E-cadherin deals, indeed, with cell adhesion, stabilizing the structure of tissues [30]. The downregulation of E-cadherin alters cellular architecture, weakening cellular junctions and favoring tumoral invasion [31]. Upon E-cadherin downregulation, β-catenin is free to translocate into the nucleus, thus exerting its transcriptional activity [32]. One of its target genes is ADAM10, a metalloprotease that, reducing cell adhesion, favors cell migration, while promoting the translocation of β-catenin to the nucleus [31]. Slug [33] or Twist [34], which play important roles in the destabilization of cell junction, besides pro-invasive factors that favor both the motility and loss of polarity of epithelial cells, such as laminin-5γ2 [31,35], are also targets of Wnt/β-catenin.

### 2.2. The Non-Canonical Wnt Signaling Pathway

The main non-canonical Wnt signaling pathways are the Wnt/Planar cell polarity (PCP) and Wnt/Ca^2+^ signaling pathways [36]. Wnt4, Wnt5a, Wnt5b, Wnt7a, Wnt7b, and Wnt11 activate non-canonical pathways by binding the Frizzled receptors. In particular, in the Wnt/Calcium (Ca^2+^) pathway, the activation of the Fzd/Dvl complex enables phospholipase C γ to convert phosphatidylinositol 4,5-bisphosphate (PIP2) into diacylglycerol (DAG) and inositol 1,4,5-triphosphate (IP3), leading to the increased release of intracellular Ca^2+^. The release of Ca^2+^ causes, in turn, the activation of calcium-dependent kinases, including Ca^2+^-dependent phosphatase calcineurin (CaN), Ca^2+^-calmodulin dependent kinase II (CAMKII), or protein kinase C (PKC) [37]. Once activated, CaMKII phosphorylates TGFβ-activated kinase 1 (TAK1), inducing Nemo-like kinase (NLK) activation. In turn, the TAK1/NLK pathway is able to counterbalance the canonical Wnt/β-catenin pathway [38]; CaN, instead, induces the nuclear factor of activated T-cells (NFAT) family proteins to move into the nucleus, increasing their transcriptional activity. In the Wnt/PCP pathway, the activated Fzd/Dvl complex determines the activation of the Rho family small GTPases RhoA, Rac and Cdc42 [39]. Cdc42 and Rac induce the activation of the c-Jun N-terminal kinase (JNK) signaling, leading to the activation of the activating protein-1 (AP-1) complex [40], while RhoA activates ROCK (Rho-associated kinase) [41]. These pathways mainly modulate cell motility and polarity [42,43].

## 3. Molecular Players Involved in the Wnt/β-Catenin-Mediated Anticancer Activity of Metformin

### 3.1. DVL3

Upregulation of Wnt signaling is a strong cancer-driving force for multiple types of malignancies [36]. In most cases, Wnt signaling upregulation is due to loss-of-function mutations in the APC gene or stabilizing mutations in the β-catenin gene, both resulting in β-catenin accumulation. These events account for 95% of incidences of colorectal cancer (CRC) but are often also found in tumors of other origin, such as the liver [44], kidney [45], ovary [46], prostate [47], brain [48], endometrium [49], thyroid [50], and lung [51]. However, changes in the expression and/or function of any of the components of the Wnt signaling pathway might contribute to the onset and progression of different types of cancer. Kwan and coworkers [52] reported a significant link between DVL3 upregulation and increased Wnt/β-catenin activity in cervical cancer. Interestingly, they were the first to demonstrate that AMPK activators, including metformin, block the growth of cervical cancer cells by interfering with the DVL3-mediated Wnt/β-catenin signaling. They showed, indeed, that the increase in proteasomal degradation was the molecular mechanism of the reduction of DVL3 induced by AMPK activators, such as metformin, evidencing the importance of DVL3 in the oncogenesis of cervical cancer and highlighting the importance of targeting DVL3 in cervical cancer via AMPK activators. The implication of AMPK activation in this process was supported by the fact that the use of an AMPK inhibitor (Compound C) was able to prevent DVL3 reduction induced by metformin [52]. The role of DVL3 in the anticancer activity of metformin was also highlighted in breast cancer in a similar study by Zou and coworkers [53], where the reduction in DVL3 levels upon metformin treatment was paralleled by the downregulation of β-catenin levels and its transcriptional targets cyclin D1 and c-Myc. Also in this study, increased AMPK-dependent proteasomal degradation of DVL3 was reported to be responsible for the downregulation of Wnt signaling by metformin. Interestingly, the inhibition of the DVL-3/Wnt/β-catenin axis by metformin with the impairment of the nuclear translocation of β-catenin was reported to play a role not only in cancer but also in the development of neural crest cells, since it interferes with the epithelial mesenchymal transition (EMT) process, which is crucial for neural cell crest fate determination [54].

### 3.2. MMP26

Matrix metalloproteinases (MMPs) play a key role in regulating embryogenesis, tissue morphogenesis and wound healing processes. In addition, the members of this family of proteins have the ability to disintegrate the components of the extracellular Matrix (ECM), favoring invasion and metastasis. Consequently, increased levels of these proteins have been described in different types of cancer. Metformin has been described to inhibit the expression of several members of the MMP family in cancer, such as MMP11 in male germ tumor cells [55], MMP9 in ovarian cancer cells and in fibrosarcoma cells [56,57], MMP2 in cholangiocarcinoma cells [58], melanoma cells [59] and endometrial carcinoma cells [60], and MMP2 and MMP9 in breast cancer cells [61] and in esophageal squamous cell carcinoma [62]. In these studies, different molecular mechanisms were described to explain the inhibitory activity of metformin in MMPs expression. However, the involvement of the Wnt pathway was not reported. Xu and coworkers [63] have recently shown that the overexpression of the matrix metalloproteinase-26 (MMP26) increased the invasiveness of chondrosarcoma cells. This is the only study to show that metformin is able to limit the effects of MMP26 on the invasion of cancer cells through the inhibition of the Wnt pathway, possibly by increasing β-catenin phosphorylation.

### 3.3. HNF4α

Chang and coworkers [64] found hepatocyte nuclear factor-4α (HNF4α) as a key component among multiple expression datasets of gastric cancer (GC) in whole-transcriptome profiles in Caucasians. They showed that the knockdown of HNF4α exerted antitumorigenic effects both in vitro and in vivo. Interestingly, HNF4α has been previously described as a downstream target of AMPKα [65,66], since it is phosphorylated in its ligand-binding domain (Ser304), thereby blocking dimerization and its binding capability to DNA. They demonstrated that metformin induces the tumor suppressing liver kinase B1 (LKB1) and AMPK in different GC cell lines. Moreover, metformin treatment reduces HNF4α expression, suggesting that the activation of AMPK may be responsible for the downregulation of HNF4α. Interestingly, metformin treatment also decreased WNT5A expression and its downstream target genes TCF1 and β-catenin in both GC cell lines and in xenograft models. Silencing of HNF4α showed similar effects, downregulating WNT5A expression and TCF/LEF signaling, thus confirming that WNT5A is a direct target of HNF4α in GC [64] and that the LKB1/AMPK/HNF4α/WNT5A axis constitutes a signaling cascade that might play a main role in GC tumorigenesis.

### 3.4. Wnt3a

Cancer stem cells (CSCs) are tumor cells with the ability of self-renewal that can induce tumor generation, recurrence and chemoresistance [67]. Several studies have showed that the Wnt/β-catenin pathway plays a crucial role in stem cells formation in cancer [68]. Zhang and Wang [69] have recently shown that metformin impaired the capability to form a spheroid. Moreover, metformin was also able to inhibit the expression of different markers of stemness in HCT116 colorectal cancer cells, such as aldehyde dehydrogenase 1 (ALDH1), epithelial cell adhesion molecule (EpCAM), Nanog and CD44. This was paralleled by the attenuation of the epithelial mesenchymal transition (EMT), as demonstrated by the reduction in vimentin, a known mesenchymal marker, and by the increase in E-cadherin, an epithelial marker. More importantly, they showed that these changes were a direct consequence of the capability of metformin to downregulate the expression of both Wnt3a and β-catenin. Treating CSCs with the Wnt3a/β-catenin agonist SKL2001 or increasing the expression of Wnt3a, besides inhibiting EMT markers, also attenuated the inhibition of the size and number of sphere cells induced by metformin. Metformin was also able to attenuate 5-fluorouracil resistance of HCT116 sphere cells, confirming what was observed in other types of cancer such as hepatocellular carcinoma [70], pancreatic cancer [71] and non-small cell lung cancer (NSCLC) [72].

### 3.5. Intracellular Acidification and ER Stress

The tumor microenvironment is characterized by a low extracellular pH that can be reduced to levels near to ~pH 5.5. Consequently, acidosis might become an important stress factor, forcing the selection and the evolution of cancer cells [73]. A property of metformin and other biguanide-type drugs resides in their capability to decrease the cellular ATP amount by blocking the activity of mitochondrial complex I (MCI). This event is usually paralleled by a compensatory increase in glycolysis [74] and the imbalance between the leakage of H+ from the extracellular acidified microenvironment to intracellular compartments, thereby determining an alteration of proton pumps function and the fall of intracellular pH. Melnik and coworkers [75] have recently shown that intracellular acidification caused by metformin inhibits Wnt signaling induced by Wnt3a, preferentially in cancer cells. In particular, the rise of intracellular acidification in association with the drop of ATP levels would cause Endoplasmic Reticulum (ER) stress, followed by the activation of the Unfolded Protein Response (UPR), a homeostatic cellular response that involves a transcriptional reprogramming of stressed cells [76,77]. DDIT3 (DNA damage-inducible transcript 3, CHOP, GADD153), one of the genes more strongly induced by the UPR, is a transcriptional repressor whose expression has been shown to be driven by metformin [78] and which is also capable of inhibiting Wnt signaling through the binding to LEF/TCF [79]. Melnik and coworkers confirmed, indeed, this evidence, also showing that upregulation of DDIT3 determines the direct inhibition of SOX4, a gene that favors metastasis in different tumors [80], by disrupting the TCF4/β-catenin activation complex and, thus, impairing the binding of TCF4 and CBP/p300 to SOX4 promoter. These events limited Wnt signaling, reducing both cancer cells’ growth and invasion. A link between ER stress, the UPR and the Wnt pathway has been also recently reported by our group [16]. Metformin was able, indeed, to inhibit β-catenin expression and transcriptional activity in endometrial cancer cells. This was associated with the upregulation of DDIT3, as described by Melnik et al. [75], but also to the downregulation of HSPA5, a key player of the UPR that also exerts antiapoptotic functions [81,82,83]. We observed, indeed, a reduction in both growth and survival of endometrial cancer cells following metformin treatment. Furthermore, metformin effects on both β-catenin and UPR protein levels/activity were not influenced by a specific inhibitor of AMPK activation, supporting the importance of molecular mechanisms independent from AMPK.

### 3.6. PPARGC1A

Peroxisome proliferator-activated receptor-gamma coactivator 1 alpha (PPARGC1A) is a transcriptional coactivator that, interacting with PPAR gamma, is able to regulate the expression of genes related to energy metabolism. PPARGC1A is involved in the progression and prognosis of different types of cancer, such as clear cell renal cell carcinoma [84], pancreatic cancer [85], breast cancer [86] and, lately, also in hepatocellular carcinoma (HCC). It has been shown, indeed, that PPARGC1A l expression is significantly reduced in HCC samples and that this represents a risk factor for overall survival of patients [87], suggesting that PPARGC1A might play an important role in HCC. Zuo et al. [88] reported that PPARGC1A acted as a tumor suppressor, inhibiting metastasis by suppressing glycolysis and suggested the involvement of the WNT/β-catenin/PDK1 axis. Zhang and coworkers [89] further investigated these aspects and found, via RNA-seq analysis, that the expression of BAMBI (bone morphogenetic protein and activin membrane-bound inhibitor), a survival-related gene target of the Wnt/β-catenin signaling pathway, was significantly upregulated in HCC cells when PPARGC1A was knocked down. Furthermore, in most of the HCC cohorts analyzed, they discovered a negative correlation between PPARGC1A and BAMBI expression. The downregulation of PPARGC1A in HCC and also resistance of HCC cells to lenvatininb were due to N6-Methyladenosine (m6A) modification of PPARGC1A mRNA by methyltransferase 3 (METTL3), a key m6A writer that they found highly expressed in HCC. Low PPARGC1A levels fail to repress BAMBI and, thus, the WNT/β-catenin pathway. Metformin was, instead, able to restore PPARGC1A expression by inhibiting METTL3 and, thus, reducing m6A modification of the protein, therefore inhibiting the WNT/β-catenin pathway.

### 3.7. Klotho

Klotho is mainly known as an antiaging gene, due to its ability to suppress senescence, oxidative stress, and inflammation. Thus, Klotho insufficiency seems to be involved in human aging and, specifically, in several aging-related diseases, including cancer [90]. There are two different forms of Klotho: a membrane-bound coreceptor form for fibroblast growth factor 23 (FGF23) [91] or a soluble form, exerting the functions of an endocrine mediator [92]. Klotho acts as an antitumor protein, blocking cancer cell proliferation and migration by modulating different signaling pathways usually involved in cancer, such as the Wnt/β-catenin and phosphoinositide 3-kinase (PI3K)/Akt pathways [93]. Klotho expression is reduced or silenced in several cancers, due mostly to epigenetic changes such as histone modifications, DNA hypermethylation at promoter sites and miRNAs activity, as already observed for other tumor suppressor genes [94]. Wang et al. showed, indeed, that the use of a demethylating agent, such as 5-azacytidine, led to increased expression of Klotho [95]. Klotho, in its soluble form, can prevent Wnt activation by sponging different Wnt ligands, such as Wnt3, Wnt1, Wnt5a, and Wnt4 [96,97]. In preclinical studies, metformin, besides other antidiabetic drugs (PPAR-γ agonists, GLP-1-based, GABA) has been reported to enhance Klotho expression [90]. Thus, metformin would be able to antagonize aberrant Wnt signaling due to deregulated production of Wnt ligands.

### 3.8. miRNAs

Metformin has been recognized to be effective in the prevention and treatment of cancer through multiple mechanisms, including micro-RNA (miRNA) regulation. Metformin is able, indeed, to modify miRNAs expression, thereby affecting specific downstream pathways. In particular, metformin can exert its anticancer effects by inhibiting the expression of oncogenic miRNAs and/or by upregulating miRNAs that display tumor suppressor activity, as extensively reviewed by Alimoradi et al. [98]. Oncogenic miR-21 has been shown to be upregulated in different types of cancer, such as breast, colorectal, renal, and skin cancer [98]. It has been described that metformin can inhibit the expression of miR-21, thereby negatively affecting several oncogenic pathways such as the TGF-β and the PTEN/Akt pathways [98]. However, only the study of Nangia-Makker et al. [99] evidenced a link between miR-21 downregulation by metformin and the Wnt pathway. They show the effects of metformin on survival of chemo-resistant colon cancer cells that are highly enriched in CSCs/CSLCs (Cancer Stem-Like Cells). In particular, they report that metformin can act synergistically with FuOx, a combination of 5-fluoruracil and oxaliplatin, to promote apoptosis in chemo-resistant colon cancer cells HT-29 and HCT-116. Furthermore, they observed that this drug combination was also able to inhibit colonospheres formation and to enhance their destruction. Moreover, the combinatorial treatment was able to inhibit migration of CR colon cancer cells. This in vitro evidence was confirmed in vivo, since the combination of metformin and FuOX for 5 weeks was capable of inhibiting the growth of tumor xenografts obtained by implanting chemo-resistant HCT-116 and HT-29 cells in SCID mice by almost 50%, when compared with the vehicle-treated controls. These effects were associated with the variation of specific microRNAs (miRNAs). They showed, indeed, that tumor suppressor miR-145 levels were increased while oncomiR miR-21 levels were reduced following metformin treatment, alone or in combination with FuOX. miR21, in particular, was previously shown to be upregulated in colorectal cancer, where it induces stemness in chemo-resistant colon cancer cells [100]. Furthermore, over-expression of miR-21 in HCT-116 cells resulted in increased β-catenin activity [33] and c-myc levels. Thus, the capability of metformin to downregulate miR-21 and the Wnt/β-catenin signaling pathway suggests a possible role of miR-21 in targeting not yet identified key regulators of this pathway.

## 4. Conclusions and Future Perspectives

**Figure 1:**
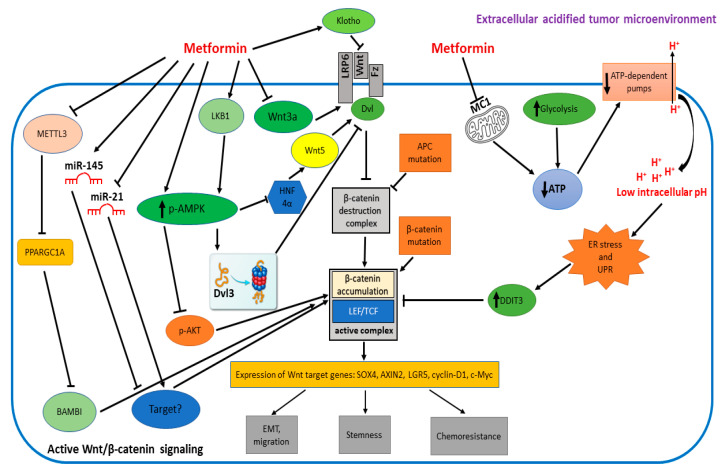
Metformin inhibits the Wnt pathway through multiple mechanisms. Wnt/β-catenin signaling can be initiated either by Wnt ligands, or by inactivating mutations of APC, or by stabilizing mutations of β-catenin, all resulting in β-catenin accumulation. β-catenin binds LEF/TCF transcription factors and induces target genes, regulating EMT, migration, stemness and chemoresistance of cancer cells. In the right section of the picture, metformin impairs ATP production and, thus, the activity of the ATP-dependent H+ pumps, leading to low intracellular pH, ER stress with UPR induction, an increase in DDIT3 and inhibition of the β-catenin/LEF/TCF complex formation. In the left section of the picture, metformin can upregulate Klotho, preventing Wnt ligand binding; inhibit directly Wnt3a; activate AMPK that can inhibit AKT, interfering with β-catenin accumulation; activate the LKB1/AMPK axis causing proteasomal destruction of Dvl3; inhibit, via AMPK, the HNF4α-dependent transcription of Wnt5; inhibit the METTL3 dependent m6A modification of PPARGC1A, causing BAMBI and β-catenin inhibition; increase tumor suppressor miR-145 and inhibit oncomiR miR-121, having inhibitory or stimulatory effects, respectively, on β-catenin activity, through still unidentified mediators.

Metformin is still the most prescribed antidiabetic agent worldwide. However, a number of different pathologic states, including cancer, have been described to benefit from metformin administration. The more extensively investigated molecular pathway is the AMPK-dependent pathway. However, in recent years, a number of other pathways have been reported to contribute to the anticancer potential of metformin. One of the more interesting and promising, from a therapeutic point of view, is the Wnt pathway. It is, indeed, often deregulated in cancer cells, where it drives important processes, such as EMT, invasion, stemness and chemoresistance. As described in this “Perspective”, metformin appears to affect the Wnt pathway at various levels (Wnt signaling complex formation, β-catenin accumulation, β-catenin transcriptional complex formation) and, often, in a cancer-specific manner (Table 1 and Figure 1). Of particular interest is the therapeutic strategy that might arise from the entrapment of cancer cells in a sort of a “Warburg Trap”, a vicious cycle determined by MC1 inhibitors such as metformin and ionophores combination, able to cause both an increase in intracellular acidification and a drop in ATP concentration, ultimately leading to apoptosis of cancer cells. Thus, a better understanding of the different molecular players that can be modulated by metformin in the Wnt pathway might be relevant in the therapy of different types of cancer.

## Figures and Tables

**Table 1:** The molecular mediators of the inhibitory activity of metformin on the Wnt pathway are shown, along with the cancer type, the molecular mechanism, and the citing references.

Mediator	Cancer Type	Mechanism	Reference
Dv13	Cervical, breast	Increased Dv13 proteasomal degradation	[53,54]
MMP26	Chondrosarcoma	Increased β-catenin phosphorylation	[57]
HNF4α	Gastric	Wnt5 downregulation	[65]
Wnt3a	Colorectal	Wnt3a and β-catenin downregulation	[70]
DDIT3	Lung, breast, colon, prostate, melanoma, glioblastoma	Inhibitory binding to LEF/TCF complex	[76,79,80]
PPARGC1A	Hepatocellular	BAMBI repression	[89,90]
Klotho	Gastric	Competitive binding to Wnt ligands	[97,98]
miR-21	Colon	Reduced β-catenin activity	[33,92]

## Data Availability

Not applicable.
